# Impact of kidney transplantation on the risk of retinal vein occlusion in end-stage renal disease

**DOI:** 10.1038/s41598-021-90765-8

**Published:** 2021-06-02

**Authors:** Jangwook Lee, Hye Rim Choe, Sang Hyun Park, Kyung Do Han, Dong Ki Kim, Kwon Wook Joo, Yon Su Kim, Eun Kyoung Lee, Un Chul Park, Hyeong Gon Yu, Hajeong Lee, Yong Chul Kim, Baek-Lok Oh

**Affiliations:** 1 Division of Nephrology, Department of Internal Medicine, Seoul National University Hospital, 101 Daehak-ro, Jongno-gu, Seoul, 03080 Republic of Korea; 2 Department of Ophthalmology, Seoul National University Hospital, 101 Daehak-ro, Jongno-gu, Seoul, 03080 Republic of Korea; 3 Department of Medical Statistics, College of Medicine, Catholic University of Korea, Seoul, Republic of Korea; 4 Department of Statistics and Actuarial Science, Soongsil University, Seoul, Republic of Korea

**Keywords:** Retinal diseases, End-stage renal disease

## Abstract

It has been known that retinal vein occlusion (RVO) is associated with chronic kidney disease, especially end-stage renal disease (ESRD). However, little is known about the effect of kidney transplantation (KT) on RVO incidence in ESRD patients. This study aimed to compare the incidence of RVO in KT recipients (n = 10,498), matched ESRD patients (n = 10,498), and healthy controls (HCs, n = 10,498), using a long-term population-based cohort. The incidence of RVO was 2.74, 5.68, and 1.02 per 1000 patient-years, for the KT group, the ESRD group, and the HCs group, respectively. Adjusted hazard ratios for RVO development compared to the HCs group, were 1.53 and 3.21, in the KT group and the ESRD group, respectively. In the KT group, multivariable regression analysis indicated that an age over 50, a Charlson Comorbidity Index score over 4, and a history of desensitization therapy were associated with an increased risk of RVO. In summary, KT recipients have a lower risk for development of RVO than ESRD patients treated with dialysis. However, the risk is still higher compared to healthy people who have normal kidney functions.

## Introduction

Retinal vein occlusion (RVO) is one of the most common causes of significant visual loss in developed countries^1^. The prevalence of RVO in hospital-based samples is variable, but prior work suggests an incidence range of 0.3–1.6% in elderly populations^2^. RVO risk is associated with systemic conditions, including hematologic abnormalities, cardiovascular disease, hypertension, diabetes mellitus (DM), smoking, dyslipidemia, and angina^3,4^.

End-stage renal disease (ESRD), the most severe form of late-stage chronic kidney disease (CKD), is associated with an enhanced mortality^5,6^. A steady increase in ESRD incidence has been observed globally, and subsequently kidney transplantation (KT), a highly effective replacement therapy for patients with ESRD, is also increasing in number^7,8^.

Recently, an association between ESRD and RVO has been reported. Comorbidities, as risk factors of RVO mentioned above, are more common in ESRD patients than in the general population^9–11^. This does not fully explain the increased risk of RVO in ESRD, however, common pathogenic mechanisms for ESRD, including vascular endothelial dysfunction and arteriosclerosis, might be contributing to RVO.

In order to prevent the development of RVO in ESRD patients, it is necessary to discover modifiable factors which may affect the RVO incidence. To our knowledge, the relationship between risk of RVO, and management of ESRD through KT, has not been investigated previously. In this study, we retrospectively recruited three patient groups: KT recipients, ESRD patients treated with renal replacement therapy, and healthy controls (HCs). We explored the hazard ratios (HRs) of RVO in these groups by analyzing the information found in the National Health Insurance Service (NHIS) Database of South Korea.

## Results

### Demographic data

Table 1 shows the baseline characteristics for the ESRD, KT, and HCs groups. The mean age of all participants at index date was 45.9 ± 10.6 years, and 59.1% of patients were male. The ESRD group and KT group exhibited a significantly higher prevalence of DM (*P* < 0.001), hypertension (*P* < 0.001), and dyslipidemia (*P* < 0.001), compared to the HCs group. Income status was classified into five grades. The ESRD group displayed a lower income status compared to the KT and HCs groups (*P* < 0.001).

**Table 1:** Baseline characteristics.

Variables	ESRD (*n* = 10,498)	KT (*n* = 10,498)	Healthy controls (n = 10,498)	*P*-value
**Age, years, *n* (%)**	45.87 ± 10.57	45.87 ± 10.57	45.87 ± 10.57	> 0.999
20–29	771 (7.3)	771 (7.3)	771 (7.3)	
30–39	2186 (20.8)	2186 (20.8)	2186 (20.8)	
40–49	3378 (32.2)	3378 (32.2)	3378 (32.2)	
50–59	3139 (29.9)	3139 (29.9)	3139 (29.9)	
60–69	974 (9.3)	974 (9.3)	974 (9.3)	
70–79	48 (0.5)	48 (0.5)	48 (0.5)	
≥ 80	2 (0.02)	2 (0.02)	2 (0.02)	
**Gender, male, *n* (%)**	6200 (59.1)	6200 (59.1)	6200 (59.1)	> 0.999
**Index year, *n* (%)**				> 0.999
2007–2009	2628 (25.03)	2628 (25.03)	2628 (25.03)	
2010–2012	3643 (34.7)	3643 (34.7)	3643 (34.7)	
2013–2015	4227 (40.26)	4227 (40.26)	4227 (40.26)	
**Income status, *n* (%)**				
Medical aid	2432 (23.17)	1496 (14.25)	261 (2.49)	
Q_1_	2895 (27.58)	2111 (20.11)	2710 (25.81)	
Q_2_	2179 (20.76)	2056 (19.58)	2570 (24.48)	
Q_3_	1751 (16.68)	2204 (20.99)	2449 (23.33)	
Q_4_	1241 (11.82)	2631 (25.06)	2508 (23.89)	
DM, *n* (%)	4231 (40.3)	4231 (40.3)	661 (6.3)	< 0.001
HTN, *n* (%)	9576 (91.22)	9576 (91.22)	1748 (16.65)	< 0.001
Dyslipidemia, *n* (%)	4441 (42.3)	5904 (56.24)	1245 (11.86)	< 0.001
**Dialysis modality, *n* (%)**				
Preemptive	0 (0)	3383 (32.23)	10,498 (100)	
Hemodialysis	7781 (74.12)	4663 (44.42)	0 (0)	
Peritoneal dialysis	2145 (20.43)	1777 (16.93)	0 (0)	
Mixed	572 (5.45)	675 (6.43)	0 (0)	
**Desensitization, *n* (%)**				< 0.001
Yes	0 (0)	1619 (15.42)	0 (0)	
No	10,498 (100)	8879 (84.58)	10,498 (100)	
**Induction, *n* (%)**				< 0.001
Not use	10,498 (100)	563 (5.36)	10,498 (100)	
ATG	0 (0)	884 (8.42)	0 (0)	
Basiliximab	0 (0)	9051 (86.22)	0 (0)	
**Maintenance, *n* (%)**				< 0.001
Not use	10,498 (100)	253 (2.41)	10,498 (100)	
Tacrolimus	0 (0)	8565 (81.59)	0 (0)	
Cyslosporine	0 (0)	1680 (16)	0 (0)	

Expressed with the number of cases (proportion). Comparison was performed using student t-test and chi-square test.

*ATG* anti-thymocyte globulin, *CI* confidence interval, *ESRD* end stage renal disease, *DM* diabetes mellitus, *HTN* hypertension, *Income status* divided into four income classes by National health insurance premium (Q_1_: lowest, Q_4_: highest), *KT* kidney transplantation.

Within the ESRD group, 74.1% of patients received hemodialysis, 20.4% of patients received peritoneal dialysis, and 5.5% of patients received both types of dialysis. In the KT recipients, 32.2% of patients had not received any type of renal replacement therapy prior to KT. The average duration for renal replacement therapy was significantly longer in the ESRD group (2.9 ± 3.0 years) compared to the KT group (2.8 ± 3.2 years). In the KT recipients, 95.6% and 15.4% of patients were treated with basiliximab and/or anti-thymocyte globulin as induction therapy, and plasmapheresis and/or rituximab as desensitization therapy, respectively, prior to KT.

### Incidence of RVO

**Figure 1:**
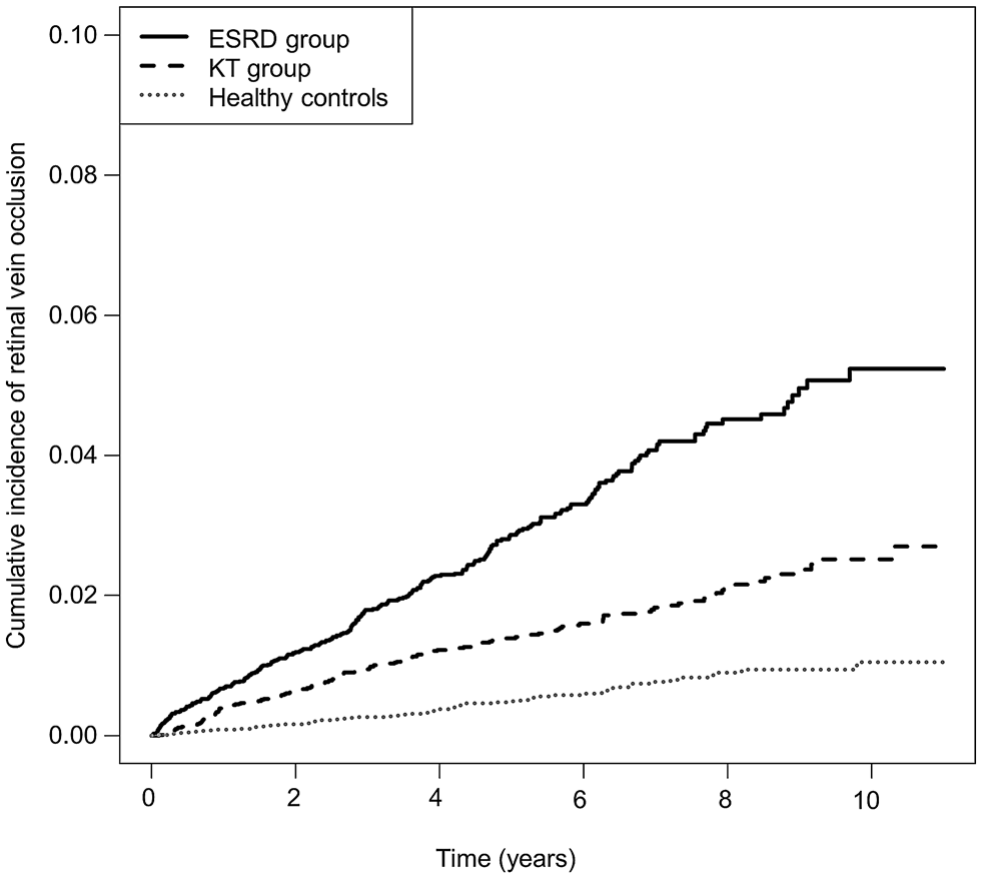
Cumulative incidence of RVO in the ESRD group, the KT group, and healthy controls. *ESRD* end-stage renal disease, *KT* kidney transplantation, *RVO* renal vein occlusion.

The cumulative incidence of RVO in the ESRD group, the KT group, and the HCs group is shown in Table 2. During the follow-up period, the incidence rates were 5.68 for the ESRD group, 2.74 for the KT group, and 1.02 for the HCs group, per 1000 patient-years respectively. Model 3, which accounted for age, sex, DM, hypertension, dyslipidemia, CCI score, and income status, indicated that the adjusted HR (aHR) for RVO development was 3.21 times greater in the ESRD group compared to the HCs group (*P* < 0.001), and 1.53 times greater in the KT group compared to the HCs group (*P* = 0.034, Fig. 1, Table 2). The number of subjects at risk of RVO among each group, stratified by time, are provided in Supplementary Table S1. A Model 3 comparison of the ESRD group and the KT group indicated that the aHR was 0.47 times lower in the KT group compared to ESRD group (*P* < 0.001). Moreover, the incidence of aHR in the ischemic and non-ischemic RVO groups was 0.44 and 0.48, respectively (Table 3).

**Table 2:** Incidence of RVO in ESRD group, KT group and healthy controls.

Outcomes	Groups	RVO	Duration		Rate	Model 1		Model 2		Model 3	
		Event	Patient-years		Per 1000 patient-years	Hazard ratio (95% CI)	*P*-value	Hazard ratio (95% CI)	*P*-value	Hazard ratio (95% CI)	*P*-value
RVO, total	ESRD	290	51,047.6		5.68	5.54 (4.22, 7.23)	< 0.001	5.71 (4.35, 7.50)	< 0.001	3.21 (2.19, 4.70)	< 0.001
	KT	164	59,847.8		2.74	2.70 (2.02, 3.60)	< 0.001	2.72 (2.03, 3.64)	< 0.001	1.53 (1.02, 2.29)	0.034
	Healthy	63	62,050.0		1.02	1 (ref)		1 (ref)		1 (ref)	
Non-ischemic RVO	ESRD	235	51,047.6		4.60	5.25 (3.90, 7.06)	< 0.001	5.49 (4.02, 7.27)	< 0.001	3.08 (2.03, 4.69)	< 0.001
	KT	135	59,847.8		2.26	2.59 (1.89, 3.56)	< 0.001	2.61 (1.91, 3.58)	< 0.001	1.49 (0.97, 2.30)	0.069
	Healthy	54	62,050.0		0.87	1 (Ref.)		1 (Ref.)		1 (Ref.)	
Ischemic RVO	ESRD	55	51,047.6		1.07	7.27 (3.60, 14.72)	< 0.001	7.53 (3.72, 15.24)	< 0.001	3.89 (1.50, 10.06)	0.005
	KT	29	59,847.8		0.48	3.33 (1.58, 7.04)	0.002	3.37 (1.59, 7.11)	0.002	1.75 (0.66, 4.66)	0.263
	Healthy	9	62,050.0		0.15	1 (Ref.)		1 (Ref.)		1 (Ref.)	

Analysis was performed using Cox regression. Model 1: not adjusted; Model 2: adjusted with age, sex; Model 3: adjusted with age, sex, diabetes mellitus, hypertension, dyslipidemia, Charlson comorbidity index, income status.

*CI* confidence interval, *ESRD* end stage renal disease, *KT* kidney transplantation, *RVO* renal vein occlusion.

**Table 3:** Hazard ratio of RVO in ESRD group and KT group.

Outcomes	Groups	Rate	Model 1		Model 2		Model 3	
		Per 1000 patient-years	Hazard ratio (95% CI)	*P*-value	Hazard ratio (95% CI)	*P*-value	Hazard ratio (95% CI)	*P*-value
RVO, total	ESRD	5.68	1 (Ref.)		1 (Ref.)		1 (Ref.)	
	KT	2.74	0.49 (0.40, 0.59)	< 0.001	0.48 (0.40, 0.58)	< 0.001	0.47 (0.29, 0.58)	< 0.001
Non-ischemic RVO	ESRD	4.60	1 (Ref.)		1 (Ref.)		1 (Ref.)	
	KT	2.26	0.50 (0.4, 0.61)	< 0.001	0.49 (0.39, 0.60)	< 0.001	0.48 (0.39, 0.60)	< 0.001
Ischemic RVO	ESRD	1.07	1 (Ref.)		1 (Ref.)		1 (Ref.)	
	KT	0.48	0.46 (0.30, 0.72)	< 0.001	0.45 (0.29, 0.70)	< 0.001	0.44 (0.28, 0.70)	< 0.001

Analysis was performed using Cox regression. Model 1: not adjusted; Model 2: adjusted with age, sex; Model 3: adjusted with age, sex, diabetes mellitus, hypertension, dyslipidemia, Charlson comorbidity index, income status.

*CI* confidence interval, *ESRD* end stage renal disease, *KT* kidney transplantation, *RVO* renal vein occlusion.

### Risk factors for RVO in KT groups

In order to determine the potential risk factors for RVO in the KT group, incidence rates were assessed by univariable and multivariable logistic regression models (Table 4). Results from univariable analysis indicate that ages greater than 60 (*P* < 0.01), CCI scores higher than or equal to 3 (*P* < 0.01), and the presence of DM (*P* < 0.001), were significantly associated with the development of RVO. Results from the multivariable regression analysis indicated that a higher age and higher CCI scores are risk factors of RVO in the KT group (*P* < 0.001). Desensitization therapy is also a potential risk factor for RVO (aHR 1.64; 95% CI 1.09–2.47). The other analyzed factors including sex, income status, DM, hypertension, dyslipidemia, and duration of dialysis, showed no relationship with RVO. Medications used for maintenance and induction of immunosuppression did not have any relationship with RVO.

**Table 4:** Risk factors analysis for RVO in KT group.

Variables	Model 1		Model 2	
	Hazard ratio (95% CI)	*P*-value	Hazard ratio (95% CI)	*P*-value
**Age, years**				
20–29	0.20 (0.74, 0.56)		0.48 (0.17, 1.36)	
30–39	0.40 (0.24, 0.65)		0.84 (0.49, 1.43)	
40–49	0.53 (0.36, 0.78)		1 (ref)	
50–59	1 (ref)	< 0.001	1.79 (1.20, 2.66)	< 0.001
60–69	1.69 (1.08, 2.57)		2.98 (1.83, 4.85)	
70–79	4.26 (1.34, 13.56)		7.89 (2.40, 5.99)	
≥ 80				
**Sex**				
Male	1 (Ref.)	0.3145	1 (Ref.)	0.560
Female	0.85 (0.62, 1.17)		0.85 (0.62, 1.176)	
**DM**				
Yes	1.96 (1.44, 2.67)	< 0.001	1.2(0.86, 1.68)	0.243
No	1 (Ref.)		1 (Ref.)	
HTN				
Yes	1.23 (0.68, 2.21)	0.495	1.05 (0.58, 1.91)	0.846
No	1 (Ref.)		1 (Ref.)	
**Dyslipidemia**				
Yes	1.20 (0.88, 1.643)	0.255	1.04 (0.75, 1.43)	0.820
No	1 (Ref.)		1 (Ref.)	
**CCI score**				
0		< 0.001		0.005
1–2	1 (Ref.)		1 (Ref.)	
3–4	1.56 (0.69, 3.51)		1.36 (0.60, 3.08)	
≥ 5	3.71 (1.73, 7.94)		2.57 (1.17, 5.65)	
**Income status**				
Medical aid	1 (Ref.)	0.7285	1 (Ref.)	0.550
Q_1_	1.04 (0.62, 1.72)		0.88 (0.52, 1.48)	
Q_2_	0.84 (0.49, 1.43)		0.71 (0.41,1.22)	
Q_3_	0.81 (0.48, 1.38)		0.66 (0.38, 1.13)	
Q_4_	1.06 (0.66, 1.72)		0.75 (0.45, 1.24)	
**Dialysis modality**				
Preemptive	0.81 (0.56, 1.17)	0.187	0.96 (0.57, 1.60)	0.107
Hemodialysis	1 (Ref.)		1 (Ref.)	
Peritoneal dialysis	1.29 (0.87, 1.92)		1.52 (1.02, 2.28)	
Mixed	0.81 (0.39, 1.68)		0.82 (0.39, 1.71)	
**Dialysis duration**				
< 1 year	1 (Ref.)	0.159	1 (Ref.)	0.809
≥ 1 year	1.25 (0.92, 1.71)		1.07 (0.67, 1.71)	
**Desensitization**				
Yes	1.55 (1.04, 2.30)	0.030	1.64 (1.09, 2.47)	0.018
No	1 (Ref.)		1 (Ref.)	
**Induction**				
Not use	0.86 (0.33, 2.25)		1.32 (0.48, 3.60)	
ATG	1 (Ref.)	0.410	1 (Ref.)	0.580
Basiliximab	1.30 (0.66, 2.56)		1.43 (0.72, 2.82)	
**Maintenance**				
Not use	0.82 (0.26, 2.57)		1.01 (0.31, 3.27)	
Tacrolimus	1 (Ref.)	0.773	1 (Ref.)	0.967
Cyslosporine	0.87 (0.58, 1.32)		0.94 (0.62, 1.43)	

Analysis was performed using univariable and multivariable Cox regression. Model 1: univariable analysis; Model 2: multivariable Cox proportional hazards regression analysis.

*ATG* anti-thymocyte globulin, *CCI* Charlson comorbidity index, *CI* confidence interval, *DM* diabetes mellitus, *HTN* hypertension, *Income status* divided into four income classes by National health insurance premium (Q_1_: lowest, Q_4_: highest), *KT* kidney transplantation, *RRT* renal replacement therapy, *RVO* retinal vein occlusion.

## Discussion

To our knowledge, this is the first study that has been conducted to explore the relationship between the incidence of RVO and KT in ESRD patients. We found that the risk for RVO in ESRD patients treated with dialysis was 3.21 times greater than in the HCs, while the KT group risk was only 1.53 times greater than in the HCs. In other words, KT could reduce the risk of subsequent RVO development in ESRD patients by about half. In the KT group, RVO development was associated with older age, higher CCI score, and a history of desensitization therapy.

The association between RVO and renal dysfunction has been explored in population-based studies. However, the definition of renal dysfunction differs based upon the study. In the Beaver Dam study, an increased level of serum creatinine was shown to be associated with the incidence of branched retinal vein occlusion (BRVO) in Caucasians^4^, but this result was not consistent with Wong’s cross-sectional study, which was conducted on 3,280 patients^12^. The Hisayama study revealed that CKD, which was defined as the presence of proteinuria and/or eGFR < 60 mL/min/1.73 m^2^, was an independent risk factor for RVO in Japanese populations^13^. In addition, extensive, longitudinal epidemiological studies, which were performed in Taiwan and South Korea, have also suggested a strong relationship between ESRD and RVO^9,11^. Similarly, our results further verify that RVO incidence is associated with ESRD.

Several mechanisms are thought to be common in the pathogenesis of RVO and ESRD. Surprisingly, the eye and kidney share many physiological features. For example, the renin–angiotensin–aldosterone hormonal cascade is found in both the kidneys as well as the eyes^14^. ESRD patients frequently experience retinal microvascular changes, such as retinal arteriolar narrowing, and arteriovenous nicking^15^. Interestingly, these retinal changes are also common in RVO patients. In addition, retinal microvascular changes are indicators of decreased renal function^14^, and predictors of RVO development. We believe that vascular endothelial dysfunction as a result of arteriosclerosis, which is the major mechanism of ESRD, contributes to retinal arteriolar changes. Vein compression, as facilitated by sclerotic arteriolar walls, may expedite thrombus formation and result in RVO. In addition, levels of proinflammatory cytokines are elevated in ESRD patients due to decreased renal elimination and increased induction by uremic toxins or oxidative stress^16,17^. The inflammation associated with these cytokines may promote the pathogenesis of RVO^18,19^. A hypercoagulable state, which is described in ESRD, can also contribute to the development of RVO. Known risk factors for RVO, such as elevated levels of prothrombin fragment, thrombin-antithrombin complex, and homocysteine^20,21^, have been described in ESRD patients^22,23^. Furthermore, repetitive hemodialysis increases the levels of oxidative stress, and stimulates inflammation and coagulation pathways^22^, which may increase the risk of RVO.

In this study, we found that in comparison to dialysis, KT can reduce the risk of RVO in ESRD patients. We also investigated the effect of dialysis duration and preemption on this result. Dialysis duration was not related to the effect of KT on RVO reduction (Table 4). Preemptive and non-preemptive transplants did not have different RVO risks (*P* = 0.469) and the RVO reduction effect by KT was still significant even when preemptive transplants were excluded from the KT group (aHR 0.50; 95% CI 0.41, 0.63). We hypothesized that KT diminished the harmful effects of uremic conditions, which are typically seen in ESRD patients on repetitive renal replacement therapy. Therefore, KT led to a protective effect by preventing RVO development. This result was in line with a well-established relationship between ESRD and cardiovascular disease (CVD), which shares common risk factors with RVO. Previous reports show that cardiovascular complications and mortality are reduced in KT recipients when compared to ESRD patients^24–26^. Interestingly, traditional risk factors (e.g., hypertension, DM, and dyslipidemia) and non-traditional risk factors (e.g., albuminuria, elevated homocysteine levels, extracellular fluid volume overload^22^, and uremia) are known to elevate the risk of cardiovascular mortality in ESRD^27^. A decreased risk of CVD in recipients has resulted from the modification of these non-traditional risk factors by KT. The common risk factors and mechanisms of pathogenesis between CVD and RVO suggest a high possibility for a protective effect of KT on RVO development in ESRD patients.

Overall, our result suggests that KT recipients had a smaller risk of RVO compared to patients with ESRD on dialysis. However, we sought to determine which KT recipients retained a higher risk of RVO development. Analysis of the KT patients indicated that older age was a significant risk factor for occurrence of RVO, as shown in previous studies^11^. Our results also revealed that the CCI score, which helps to predict cardiovascular risk in KT recipients, could be a relevant risk factor for occurrence of RVO^28,29^. In addition, immunosuppressant agents such as tacrolimus, cyclosporine, anti-thymocyte globulin and basiliximab did not affect the risk of RVO in our study. There have been some reports that cyclosporine is associated with increased hypertension and hyperlipidemia^30^ and tacrolimus is associated with an increased risk of new-onset diabetes after transplantation^31^. However, it would be possible that the risk modification for cardiovascular complication after KT as mentioned earlier ameliorated negative effect of immunosuppressant agents. Analogously, the association between the type of immunosuppression and cardiovascular death was not observed in the report investigating the effect of KT on cardiovascular death^32^. Interestingly, desensitization therapy before KT was a significant risk factor for the occurrence of RVO. Generally, desensitization therapies, including rituximab, plasmapheresis, or intravenous immunoglobulin, have been performed to reduce the risk of organ rejection in certain KT recipients, like ABO incompatible KT or human leukocyte antigen incompatible KT^33,34^. It is well known that endothelial dysfunction is induced due to up-regulation of various cytokines in sensitized KT recipients^35–37^. Therefore, we hypothesized that endothelial dysfunction and subsequent atherosclerosis, which are related to pathogenesis of RVO^38,39^, might contribute to the increased risk of RVO development in KT recipients with desensitization therapy.

Here, we present a large, population-based study to analyze KT recipients against matched ESRD patients and HCs. However, our study has some limitations. First, an ICD-10 code-dependent diagnosis can potentially lead to an inaccurate diagnosis of RVO, ESRD, and other comorbidities. Secondly, we only analyzed data from the NHIS. So detailed clinical information and aspects of the individual patients, such as vision prognosis and involvement of lesions, could not be reflected. Third, we did not distinguish subtypes of RVO, namely, central retinal vein occlusion (CRVO) and BRVO in patients. Therefore, the different pathogeneses and risk factors associated with RVO subtypes might be overlooked^40^. Forth, there was no information about such as ABO incompatibility or human leukocyte antigen incompatibility of kidney transplantation in our cohort data, so it was impossible to analyze the difference of RVO outcome according to immunologic incompatibility.

In conclusion, we found that KT recipients showed a significantly lower risk for RVO than ESRD patients treated with dialysis. Increasing age, high CCI scores, and desensitization therapy could be risk factors of RVO development in KT recipients. Therefore, although KT might be helpful to prevent RVO in ESRD patients, careful ophthalmic examination should be performed for older KT recipients who present with known comorbidities or a history of desensitization therapy.

## Methods

This study was undertaken as a part of the RESTORE (REnal tranSplanT and OculaR disEases study) project, a retrospective, longitudinal, cohort study based on the NHIS database. The primary objective of the RESTORE project was to investigate the effect of KT on the national population-based incidence and prognosis of various eye diseases, including age-related macular degeneration, RVO, and glaucoma. The study protocol was approved by the Institutional Review Board of Seoul National University Hospital (IRB No. E-1908-001-1050) and all methods were carried out in accordance with relevant guidelines and regulations. The need for participant consent was waived by the Institutional Review Board of Seoul National University Hospital because this study collected medical data which is managed anonymously.

### Data source and collection

South Korea has a compulsory single-payer health insurance system, managed by the NHIS. All healthcare providers are required to submit medical claims with information including demographics, diagnostic codes based on the International Classification of Diseases (ICD)‐10 codes^41^, procedure codes, prescription records, and healthcare facilities to the NHIS for review and reimbursement. Therefore, the NHIS claims system acts as a centralized database and provides a nationwide, population-based data source.

**Figure 2:**
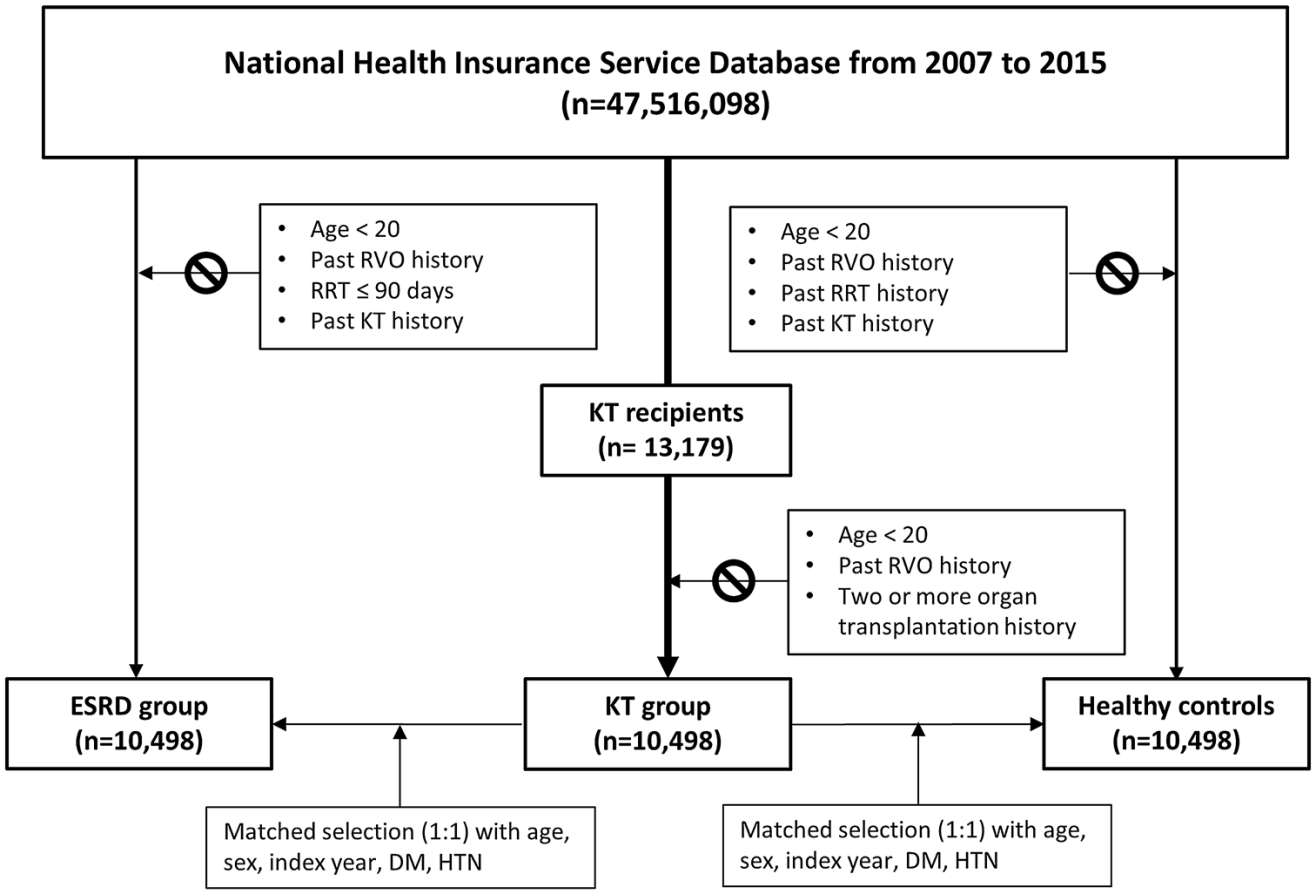
Flowchart depicting the identification of the KT group, the ESRD group, and the healthy controls. *ESRD* end-stage renal disease, *DM* diabetes mellitus, *HTN* hypertension, *KT* kidney transplantation, *RRT* renal replacement therapy, *RVO* renal vein occlusion.

In this study, subjects were recruited from the NHIS database from the year 2007 to 2015. Using specific codes for kidney transplantation (R3280 [KT, ICD-10 code], V005 [KT related treatment, V code for Korean rare incurable diseases]) we were able to identify 13,179 KT recipients. Patients were excluded if they (1) were less than 20 years old, (2) had previously been diagnosed with RVO, and (3) had received two or more organ transplantations. After exclusions, we analyzed 10,498 KT recipients. The ESRD group consisted of patients identified by the ICD-10 codes (N18-19, Z49, Z94.0, Z99.2) and the procedural codes of renal replacement therapy (hemodialysis [O7011-7020, V001] or peritoneal dialysis [O7071-7075, V003]). Patients were required to have been diagnosed with ESRD for more than 3 months, and had not previously received a KT. By considering age, sex, inclusion year, underlying hypertension and DM, the ESRD patients were matched with the KT recipients. Individuals without history of renal replacement therapy, including KT and RVO history, were selected as HCs and were matched with KT patients by age, sex, inclusion year, and history of underlying hypertension and DM (Fig. 2).

Demographic characteristics including age, sex, income status, type of dialysis, duration of dialysis, time of KT, type of induction therapy, history of desensitization therapy, and the Charlson Comorbidity Index (CCI) were identified for each patient^42^. Preemptive KT was defined as KT performed within 3 months of dialysis or without dialysis. Hypertension was identified by a previous diagnosis of hypertension (I10-I15, I159, I150, I1522, I151, I1528) or the prescription of antihypertensive drugs more than twice within one year before KT. DM was defined by a previous DM diagnosis (E109, E119, E139, E149, E101, E111, E131, E141, E105, E115, E135, E145) or prescription of one or more oral hypoglycemic agents or insulin. Dyslipidemia was defined by a diagnosis of dyslipidemia (E78) or a prescription of lipid-lowering agents. Among the prescription lists, information regarding immunosuppressant agents such as tacrolimus, cyclosporine, and steroids, which were licensed in South Korea during the follow-up, was collected separately.

### Study outcomes

The main goal was to determine the incidence of RVO in KT recipients compared to that in ESRD patients and in HCs. The study population was followed up to the date of RVO diagnosis or until December 31, 2017, whichever came first. RVO was confirmed by ICD-10 code H43.8. We subdivided the RVO patients into two subgroups: ischemic and non-ischemic. Ischemic RVO was operationally defined as those who needed scatter laser photocoagulation (S5160, S5161) because of a large non-perfusion area, or who underwent vitrectomy (S5121, S5122) within one year after RVO diagnosis. Otherwise the case was defined as non-ischemic RVO.

### Statistical analysis

Descriptive characteristics are presented as mean ± SD, median (IQR), number, or frequency (%). The chi‐square test was used to determine differences in the proportion of categorical variables, and analysis of variance (ANOVA) was used to evaluate differences between the means of continuous variables. Incidence rates of RVO are expressed as events per 1000 patient-years.

The cumulative incidence of RVO was analyzed using Kaplan–Meier estimates. Log-rank test was used to compare differences among groups. HR and 95% confidence interval (CI) values were analyzed using a Cox proportional hazards analysis with multivariable-adjustment. Model 1 presented the results of an unadjusted univariable analysis, and Model 2 was adjusted for age and sex. Model 3 further adjusted Model 2 for hypertension, DM, dyslipidemia, CCI, and income status. All analyses were performed with the SAS 9.4 program (SAS Institute, Cary, NC). *P* values < 0.05 were considered statistically significant.

## Supplementary Information

Supplementary Information.
