# Supplementary material for: Impact of kidney transplantation on the risk of retinal vein occlusion in end-stage renal disease
Source: Sci Rep. 2021 Jun 2;11:11583. doi: 10.1038/s41598-021-90765-8 (PMC8172893; doi:10.1038/s41598-021-90765-8)
Supplement: Supplementary file 1 — Supplementary Information. [file 41598_2021_90765_MOESM1_ESM.docx]

**Supplementary Table S1. The numbers of subjects at risk of RVO in ESRD group, KT group and healthy controls**

| **Group** | **Years** | | | | | | | | | | | | |
| --- | --- | --- | --- | --- | --- | --- | --- | --- | --- | --- | --- | --- | --- |
|  | **0** | **1** | **2** | **3** | **4** | **5** | **6** | **7** | **8** | **9** | **10** | **11** |  |
| ESRD | 10,498 | 9,883 | 9,263 | 7,505 | 5,954 | 4,590 | 3,348 | 2,314 | 1,592 | 933 | 401 | 4 |  |
| KT | 10,498 | 10,235 | 10,130 | 8,631 | 7,210 | 5,882 | 4,536 | 3,341 | 2,395 | 1,497 | 676 | 11 |  |
| Healthy Controls | 10,498 | 10,451 | 10,421 | 8,941 | 7,523 | 6,169 | 4,782 | 3,524 | 2,548 | 1,607 | 738 | 10 |  |

ESRD: end stage renal disease; KT: kidney transplantation; RVO: renal vein occlusion
